# Editorial: Advancing our understanding of the impact of dynamics at different spatiotemporal scales and structure on brain synchronous activity

**DOI:** 10.3389/fnetp.2022.1038239

**Published:** 2022-10-06

**Authors:** Thanos Manos, Chris G. Antonopoulos, Antonio M. Batista, Kelly C. Iarosz

**Affiliations:** ^1^ Laboratoire de Physique Théorique et Modélisation, CY Cergy Paris Université, CNRS, Cergy-Pontoise, France; ^2^ Equipes Traitement de l’Information et Systèmes (ETIS), CNRS, Cergy Paris Université, Cergy-Pontoise, France; ^3^ Department of Mathematical Sciences, University of Essex, Colchester, United Kingdom; ^4^ Department of Mathematics and Statistics, State University of Ponta Grossa, Ponta Grossa, Brazil; ^5^ Science Graduated Program, State University of Ponta Grossa, Ponta Grossa, Brazil; ^6^ Oscilations Control Group, Institute of Physics - University of São Paulo, University City, São Paulo, Brazil; ^7^ Exact Sciences, Natural and Engineering, University Center FATEB, São Paulo, Brazil; ^8^ Chemical Engineering Graduate Program, Federal University of Technology—Paraná, Paraná, Brazil

**Keywords:** brain dynamics, neural networks, plasticity, synchronization, collective activity, neuron models

In the last decades, complex networks have been used extensively in neuroscience and other fields by employing networks to model interactions among system’s components (Sporns et al., 2005). Moreover, there is cross-breeding between mathematical neuroscience and clinical neuroimaging techniques (Popovych et al., 2019). This interaction helps improve existing dynamical models or derive new ones which can then reproduce more accurately healthy and pathological brain activity. Consequently, they can help better detect underlying causes, and lead to testing treatment strategies in simulated environments (Jirsa et al., 2017). The human brain is a prime example of a complex system that can self-organise into different emergent states, crucial for healthy, abnormal functioning, and cognition (see e.g. (Majhi et al., 2019), for a recent review). These originate from certain types of synchronous neural activity. Brain disorders such as Parkinson’s disease, epilepsy, etc. are associated with abnormal neural synchrony.

A common research theme in the study of complex networks is that of the determination of the role and impact of network topology on the emerging properties due to interactions and dynamics. How structural properties in brain regions affect neural dynamics or how do they differ in healthy and diseased subjects? How different types of plasticity affect the dynamics of neural activity in a network?

This Research Topic contributes to better understand the impact of dynamics at different spatiotemporal scales, structure and plasticity on brain synchronous activity. To this end, it hosts interdisciplinary analytical and computational works from different fields, such as from complex systems, biophysics, systems biology, and computational neuroscience.

The first paper, by Liu et al., aims to better understand the effect of the amyloid *β* peptide (A*β*) which is hypothesized to be the major factor driving Alzheimer’s disease (AD) pathogenesis. To this end, the authors derived and investigated a concise mathematical model for A*β*-mediated multi-pathway astrocytic intracellular Ca2+ dynamics. They investigated several interventions, such as ion channel blockers or receptor antagonists and demonstrated that a “combination therapy” targeting multiple pathways simultaneously is more effective towards the treatment of AD.

Cambell et al. investigated the fractal dynamics of blood oxygen level-dependent (BOLD) signals during naturalistic conditions. They implemented fractal analysis to quantify scaling behavior using the Hurst exponent (*H*) in data from the Human Connectome Project to compare *H* values across movie-watching and rest. Their results showed that movie-watching induces fractal signal dynamics and markedly different than dynamics observed during conventional tasks.

van der Vlag et al. introduced a new computational tool called RateML that enables users to generate whole brain network models from a succinct declarative description, in which the mathematics of the model are described without specifying how their simulation should be implemented. With RateML, the end user describes the model’s mathematics once and generates and runs code for different languages/platforms, targeting both CPUs for fast single simulations and GPUs for parallel ensemble simulations (e.g. explore broader parameter fitting workflows, support studies on larger cohorts, etc).

Lei et al. introduced a new type of burst-oscillation mode (BOM), i.e. an alternating transition between two distinct phases (one with multiple short spikes and another with a long interval). BOM was derived by extensively investigating the response dynamics of a one-dimensional (1D) paced excitable system with unidirectional coupling. These findings may facilitate a deeper understanding of bursts in nature and will have a useful impact in related fields.

Shi et al. proposed a deep attributed network representation learning with community awareness (DANRL-CA) framework and two variants, i.e., DANRL-CA-AM and DANRL-CA-CSM, which incorporate the community information and attribute semantics into node neighbours with different methods. They designed a neighbourhood enhancement autoencoder module to capture the 2-step relations between node pairs. They conducted comparisons for node classification and link prediction and found that DANRL-CA-CSM can more flexibly coordinate the role of node attributes and community information in the process of network representation learning, and shows superiority in the networks with sparse topological structure and node attributes.

Madadi Asl et al. employed a computational model to show that inhibitory spike-timing-dependent plasticity (iSTDP) at pallido-subthalamic synapses can account for pathological strengthening of pallido-subthalamic synapses in Parkinson’s disease (PD) by further promoting correlated neuronal activity in the globus pallidus (GPe) - subthalamic nucleus (STN) network. Their results may shed light on how abnormal reshaping of GPe-STN connectivity by synaptic plasticity during parkinsonism is related to PD pathophysiology and contribute to the development of therapeutic brain stimulation techniques targeting plasticity-induced rewiring of network connectivity.

Zheng et al. studied the emergence of spatiotemporal patterns in a general networked Hindmarsh-Rose (HR) model. Furthermore, they investigated stability properties, namely they obtained conditions leading to Turing instability in networks without delays and showed that there is a difference between the collected current and the outgoing current affecting the neuronal activity, which is relevant in the generation mechanism of short-term memory.

Li et al. investigated the dynamics of networks of identical coupled oscillators in a setting where coherent oscillations coexist with incoherent ones, the so-called chimera state. They showed that stable and breathing chimera states in the original two coupled networks typically have very small basins of attraction. Then, they studied the emergence of chimera states by stimulating brain regions and quantified it using the order parameter and chimera index. These two indices were found to be weakly and negatively correlated.

## References

[B1] Jirsa V. K. Proix T. Perdikis D. Woodman M. M. Wang H. Gonzalez-Martinez J. (2017). The virtual epileptic patient: Individualized whole-brain models of epilepsy spread. Neuroimage 145, 377–388. 10.1016/j.neuroimage.2016.04.049 27477535

[B2] Majhi S. Bera B. K. Ghosh D. Perc M. (2019). Chimera states in neuronal networks: a review. Phys. Life Rev. 28, 100–121. 10.1016/j.plrev.2018.09.003 30236492

[B3] Popovych O. V. Manos T. Hoffstaedter F. Eickhoff S. B. (2019). What can computational models contribute to neuroimaging data analytics? Front. Syst. Neurosci. 12, 68. 10.3389/fnsys.2018.00068 30687028 PMC6338060

[B4] Sporns O. Tononi G. Kotter R. (2005). The human connectome: a structural description of the human brain. PLoS Comput. Biol. 1, e42. 10.1371/journal.pcbi.0010042 16201007 PMC1239902

